# Tender coconut water an economical growth medium for the production of recombinant proteins in *Escherichia coli*

**DOI:** 10.1186/1472-6750-13-70

**Published:** 2013-09-02

**Authors:** Narendrakumar Sekar, Soumya Kariyadan Veetil, Muniasamy Neerathilingam

**Affiliations:** 1 Protein Technology Core, Centre for Cellular and Molecular Platforms, NCBS Campus, GKVK, Bellary Road, Bangalore 560 065, Karnataka, India

**Keywords:** Coconut water, Growth media, *E.coli*, *Pichia pastoris*, Protein expression, Natural media

## Abstract

**Background:**

*Escherichia coli* is most widely used prokaryotic expression system for the production of recombinant proteins. Several strategies have been employed for expressing recombinant proteins in *E.coli*. This includes the development of novel host systems, expression vectors and cost effective media. In this study, we exploit tender coconut water (TCW) as a natural and cheaper growth medium for *E.coli* and *Pichia pastoris.*

**Result:**

*E.coli* and *P.pastoris* were cultivated in TCW and the growth rate was monitored by measuring optical density at 600 nm (OD_600nm_), where 1.55 for *E.coli* and 8.7 for *P.pastoris* was obtained after 12 and 60 hours, respectively. However, variation in growth rate was observed among TCW when collected from different localities (0.15-2.5 at OD_600nm_), which is attributed to the varying chemical profile among samples. In this regard, we attempted the supplementation of TCW with different carbon and nitrogen sources to attain consistency in growth rate. Here, supplementation of TCW with 25 mM ammonium sulphate (TCW-S) was noted efficient for the normalization of inconsistency, which further increased the biomass of *E.coli* by 2 to 10 folds, and 1.5 to 2 fold in *P.pastoris.* These results indicate that nitrogen source is the major limiting factor for growth. This was supported by total nitrogen and carbon estimation where, nitrogen varies from 20 to 60 mg/100 ml while carbohydrates showed no considerable variation (2.32 to 3.96 g/100 ml). In this study, we also employed TCW as an expression media for recombinant proteins by demonstrating successful expression of maltose binding protein (MBP), MBP-TEV protease fusion and a photo switchable fluorescent protein (mEos2) using TCW and the expression level was found to be equivalent to Luria Broth (LB).

**Conclusion:**

This study highlights the possible application of TCW-S as a media for cultivation of a variety of microorganisms and recombinant protein expression.

## Background

An ideal growth medium for microbes requires many macro and micronutrients in appropriate proportion for optimal growth and metabolism. Carbon and nitrogen are the major sources for microbial growth, while trace elements like sulphur, phosphorus, vitamins etc., are micronutrients. Further, natural media has long served as a source for microbial propagation. Urine, meat extract, potato pieces, sprouted barley, soya flour etc., are some pioneer media employed for microbial growth [1,2]. Even in the present days, yeast extract, beef extract, casein, are among the major ingredients of commercially available culture media [2]. Being readily available and serving as a rich source of essential nutrients for the growth of microbes, such natural components are widely used in chemically undefined media. However, these involve tedious procedures like extraction of nutrients, their maintenance, sterilization and supplementation to meet the requirements for microbial growth and development.

Tender coconut water (TCW), liquid endosperm present in the cavity of the coconut fruit consists of nutrients which comprises of 95.5% water, 4% sugars, 0.1% fat, 0.02% calcium, 0.01% phosphorous, 0.5% iron, considerable amounts of amino acids, mineral salts, vitamin B complex, vitamin C and cytokines etc. [3]. Because of the rich nutrient content in coconut water, it was noted for its wide applications in plant tissue culture, growing fungus and other microbes [4-6]. In few such studies, coconut water was used as complete growth media for *Rhodotorula glutinins*[7]. Whereas, Unagul *et al*., demonstrated the supplementation of coconut water to yeast extract-diluted seawater medium for the production of docosahexaenoic acid (DHA), which was 50% higher than that of non-supplemented media [8]. Similarly, coconut water was used as a raw material for supplementation of carbon and nitrogen in MRS- sucrose media for the production of exopolysaccharide (EPS) by *Lactobacillus confusus* to reduce the cost of fermentation medium [9]. In another study, Prabakaran *et al.,* reported the production of δ endotoxin, an endogenous protein of *Bacillus thuringiensis* var. *israelensis*, (a biological control agent against mosquitoes) using coconut water as a growth medium [10]. Although the above studies focused on growth of microbes and endogenous protein production in coconut water, its application in recombinant protein production has not been demonstrated before.

In recent years, the therapeutic application of recombinant proteins has been increased immensely. Many industries are producing large quantities of recombinant proteins and the need for expression media devoid of any toxin has increased subsequently. TCW is plant derived and deficient in endotoxins which are present in other commercially available media. Hence, this isotonic beverage could be a safe alternative medium for therapeutic protein production. Since, TCW is naturally sterile, the preparation and sterilization is fairly convenient in comparison to conventionally used media. It is economical and abundantly available throughout the year (especially tropical and coastal areas), thereby contributing to its feasibility as a potential microbial growth medium.

## Methods

### Strains and plasmids

*E.coli* strains such as BL21 (DE3), BL21 (DE3) pLysS were used for protein expression studies whereas, *E.coli* C41 (DE3) and *P.pastoris* GS115 were used for growth studies. All the strains were procured from Invitrogen™, USA. Three different constructs were used namely; maltose binding protein (MBP), a fusion of MBP with tobacco etch virus protease (MBP-TEV) and monomeric variant of photo switchable fluorescent Eos proteins (mEos2). pMAL-c5× for expressing MBP, pMAL-c5× harboring TEV for expressing MBP-TEV fusion and pRSET-A harbouring mEos2 were used in this study. TEV was synthesized by GeneScripts, USA and mEos2 was a kind gift from Dr. Satyajit Mayor, NCBS, Bangaluru, India.

### Preparation of TCW media and agar plates

**Figure 1:**
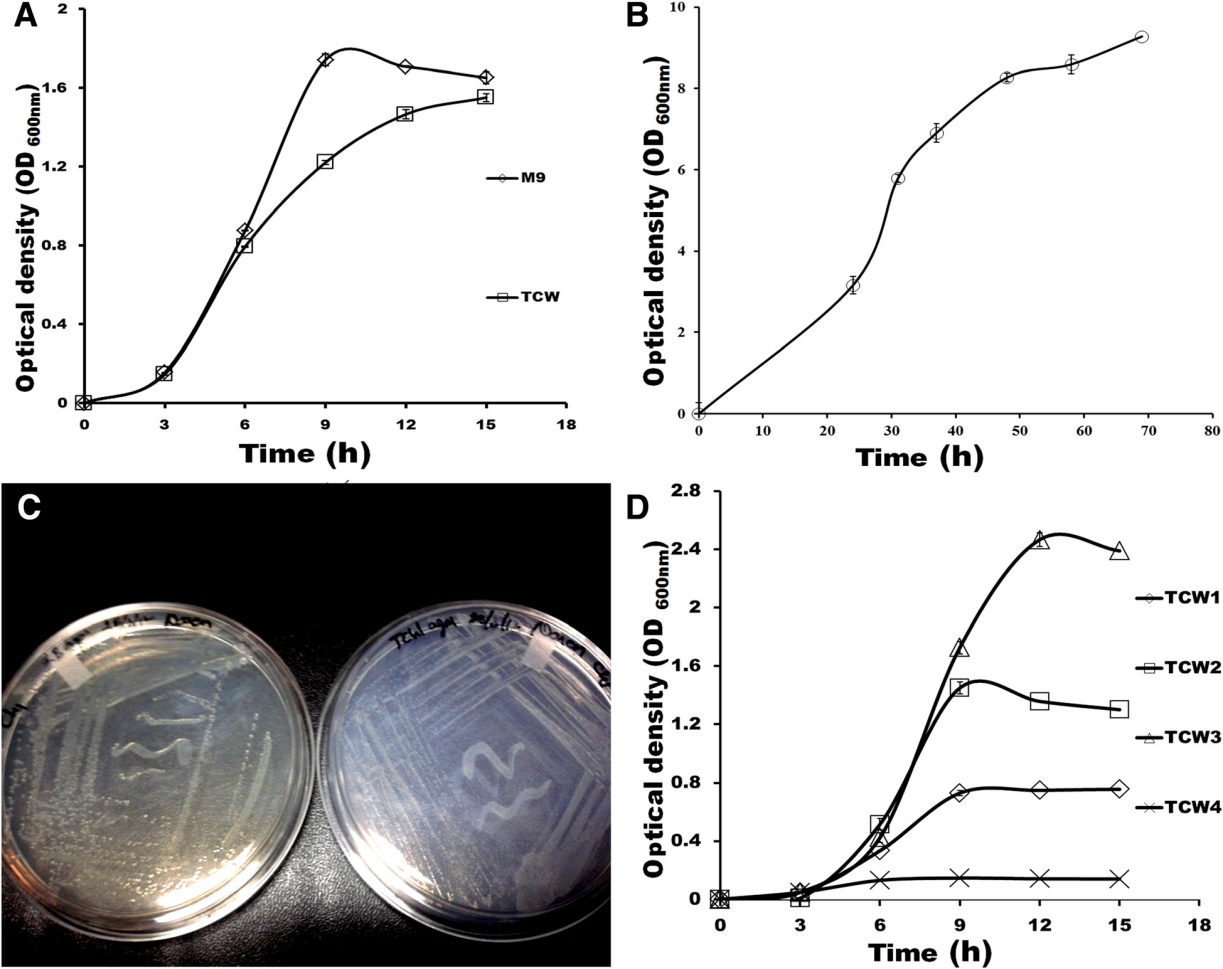
**Growth curve of *E.coli *and *P.pastoris *in TCW.** Growth curve of **(A)***E.coli* C41 in TCW and M9 media **(B)***P.pastoris* in TCW **(C)** Growth of *E.coli* C41 on LB agar plate (left) and TCW agar plate (right). **(D)** Growth curve of C41 in different TCW collected from different places. The experiments were performed in triplicates and SD has been incorporated.

Tender coconut fruits were obtained from different places in Bengaluru, India. Part of mesocarp and endocarp were removed to expose the surface of endosperm. Then, coconut water (TCW) was extracted and centrifuged at 4000 rpm for 20 minutes to separate particulate matter, followed by filter sterilization with 0.22 μm filter (*Millex*® filter units –*Millipore*). TCW agar plates (Figure 1C) were prepared by mixing 25 ml of filter sterilized TCW with 25 ml of 4% agar (autoclaved) and microwaved for 30–60 seconds.

### Supplementation of TCW (TCW-S) with carbon, nitrogen and other salts

Stock solutions of 1M (NH_4_)_2_SO_4_, 1M Na_2_SO_4_, 1M KH_2_PO_4_, 1M MgSO_4_ and 80% glycerol, 20% glucose, 20% lactose and 1% amino acids cocktail (1% of each of 17 amino acids excluding cysteine, methionine and tyrosine) were sterilized by autoclaving except glucose, MgSO_4_ and amino acids which were filter sterilized by 0.22 μm filter. These compounds were supplemented to TCW at a final concentration of; 25 mM (NH_4_)_2_SO_4_, 5 mM Na_2_SO_4_, 50 mM KH_2_PO_4_, 2mM MgSO_4_, 0.8% glycerol, 0.2% glucose , 0.5% lactose and 0.36% amino acid cocktail (Table 1).

**Table 1:** List of tested supplements: 1M stock solutions were prepared by dissolving the salts in water

**Chemical name**	**Stock solution concentration**	**Final concentration**
(NH4)2SO4	1 M	25 mM
Na2SO4	1 M	5 mM
KH2Po4	1 M	50 mM
MgSO4	1 M	2 mM
Glycerol	80%	0.8%
Glucose	40%	0.2%
Lactose	20%	0.5%
Amino acid mixture^1^ (− cys,-met,-tyr)	10 mg/ml each of 17 amino acids^1^	0.36%

The above mentioned reagents were prepared as described by Neerathilingam and Markley [21].

^1^Amino acid cocktail without cystein, methionine and tyrosine was prepared**.**

### Estimation of total carbohydrate, nitrogen and other metabolites in TCW

The concentration of amino acids and metabolites were estimated using LC-MS/SRM (Liquid Chromatography-Mass Spectrometry/Selected Reaction Monitoring) method. All amino acids, acetone, formic acid were obtained from Sigma-Aldrich (Bangalore, India). The corresponding deuterated internal standards (ISTD) were obtained from CDN isotopes (Quebec, Canada). Acetonitrile and water used for the chromatography were obtained from Thermo Fisher Scientific. 10 ng of ISTD mix of all amino acids were spiked with 10 μL of TCW and amino acids were extracted by precipitating the proteins using 200 μL of acetone (0.1% FA). It was then vortexed, centrifuged (13000 rpm, 5min) and the supernatant was dried using speed vacuum. The derivatization of amino acids was done in the similar way using the 6-aminoquinolyl-N-hydroxysuccinimidyl carbamate as previously published procedure [11]. The analysis was done using the LC-MS system (LC-Agilent 1290 infinity series, MS- Thermo Fisher TSQ vantage). The single major product ion of m/z 171 was used for the SRM transitions. LC conditions [Solvent system A-water (0.1% FA), B-acetonitrile (0.1% FA), flow-200 μL/min, column- C-18 (2.1 × 100 mm, 1.8 μm, Phenomenex), gradient- 0 to 3 min- 2% B, 3–20 min-20% B, 20 to 25 min- 35%, 25 to 27-80% B, 27–30 min-2% B]. MS conditions [spray voltage-3700 V, ion transfer capillary temperature 270°C, source temperature 30°C, sheath gas 20, auxiliary gas 10 (arbitrary units), collision gas-argon, S-lens voltage and collision energy were optimized for individual amino acids, scan time-50 millisec, ion polarity is positive). The final relative quantification was done based on the area under the curve of individual amino acid ISTDs.

Total nitrogen in TCW was estimated by conventional Kjeldahl method [12]. Total carbohydrate was measured by phenol sulphuric acid method as described by Dubious *et al*., [13].

### Inoculum preparation and culture conditions

For growth studies, *E.coli* C41 (DE3) was streaked on LB-agar plates and *P.pastoris* GS115 on YPD (Yeast (1%) Peptone (2%) Dextrose (2%) medium) agar plate, then incubated at 37°C and 30°C for overnight, respectively. A loop-full of colonies were inoculated into 10 ml of TCW or M9 (1 g NH_4_Cl, 3 g KH_2_PO_4_, 6 g Na_2_HPO_4_, 4 g glucose, and 1 ml of 1M MgSO_4_/L) minimal medium (wherever applicable) and incubated for overnight at 37°C for *E.coli* and 30°C for *P.pastoris* (primary culture) with 200 rpm. Primary culture (*E.coli*: ~1.5 × 10^8^, *P.pastoris*: ~1.5 × 10^6^ cells) was then inoculated into 250 ml flask containing 50 ml of TCW/TCW-S/M9 (wherever applicable) and the growth was monitored by measuring OD_600nm_. All the experiments were performed in triplicates and mean standard deviation was calculated.

To study protein expression in TCW, constructs of MBP and MBP-TEV fusion were transformed into BL21 (DE3) and mEos2 into BL21 (DE3) pLysS. One percent of overnight culture was inoculated into 250 ml flask containing 50 ml of TCW, TCW-S (supplemented) and LB media containing ampicillin (100 μg/ml) followed by incubation at 37°C with 200 rpm. The cultures were induced with IPTG (0.4 mM for MBP, MBP-TEV and 0.1 mM for mEos2) at OD_600nm_ of 0.5-0.6 and incubated at 30°C for 5 hours. Further, samples were harvested and centrifuged at 4,000 rpm for 15 minutes at 4°C. Expression level was checked on 12% SDS-PAGE.

## Results and discussion

### Growth of *E.coli* and *P.pastoris* in TCW and TCW-S

TCW being rich in nutrients contains all the essential components that are required for the growth of microorganisms. In order to demonstrate its use as a complete media, *E.coli* was grown in TCW and a maximum OD_600nm_ of 1.55 was noted after 12 hours. The growth rate was compared to that of conventional M9 media, which showed OD_600nm_ maximum of 1.70 (Figure 1A) after 12 hours of incubation.

*E.coli* is known to grow at pH 4.0 to 8.0 [14]. However, the optimum pH is reported as 6.5- 7.0 [14]. We noted the pH of TCW as 4.7 ± 0.2. In this regard, a 50 ml sample was pH adjusted to 7.0 and growth was monitored. After overnight incubation, the pH was reduced and found to be equivalent to that of unadjusted TCW, with no difference in growth rate. Hence, all experiments were performed without adjusting pH. Nevertheless, to achieve optimal condition, pH needs to be monitored and maintained. We also used TCW as a complete growth media for *P.pastoris* where a maximum OD_600nm_ of 8.7 was noted after 64 hours (Figure 1B). In addition, TCW agar plates were used for plating *E.coli*, where the colonies appeared to be similar in morphology to that of LB agar plates (Figure 1C).

Since TCW is a naturally occurring liquid, its chemical composition varies from coconut to coconut [15]. To validate and demonstrate the effect of this variation on growth, biomass of *E.coli* in different TCW samples were studied, which ranged from OD_600nm_ of 0.15-2.5 after 12 hours of incubation (Figure 1D). This indicates that nutrients present in TCW vary with each coconut which could presumably be due to; the maturity, location and variety of coconut fruit, thus influencing its chemical profile. For instance, sugars like glucose and fructose are higher in young coconut (TCW) [15,16] whereas, sucrose is the predominant sugar in mature coconut water [17,18]. Kuberski *et al*., made similar observations where, sugar content in coconut water was identified as glucose, sucrose and fructose in the proportion of approximately 50%, 35% and 15%, respectively [19]. In another study, Vigliar *et al.*, [3] demonstrated that the proportion of these sugars varied depending upon the stage of maturation of the coconut fruit i.e. glucose varies from 34% to 45%; sucrose from 53% to 18% and fructose from 12% to 36%. Similarly, other nutrient components of TCW vary significantly with degree of maturation like potassium, chloride, iron and sulphur [20].

**Figure 2:**
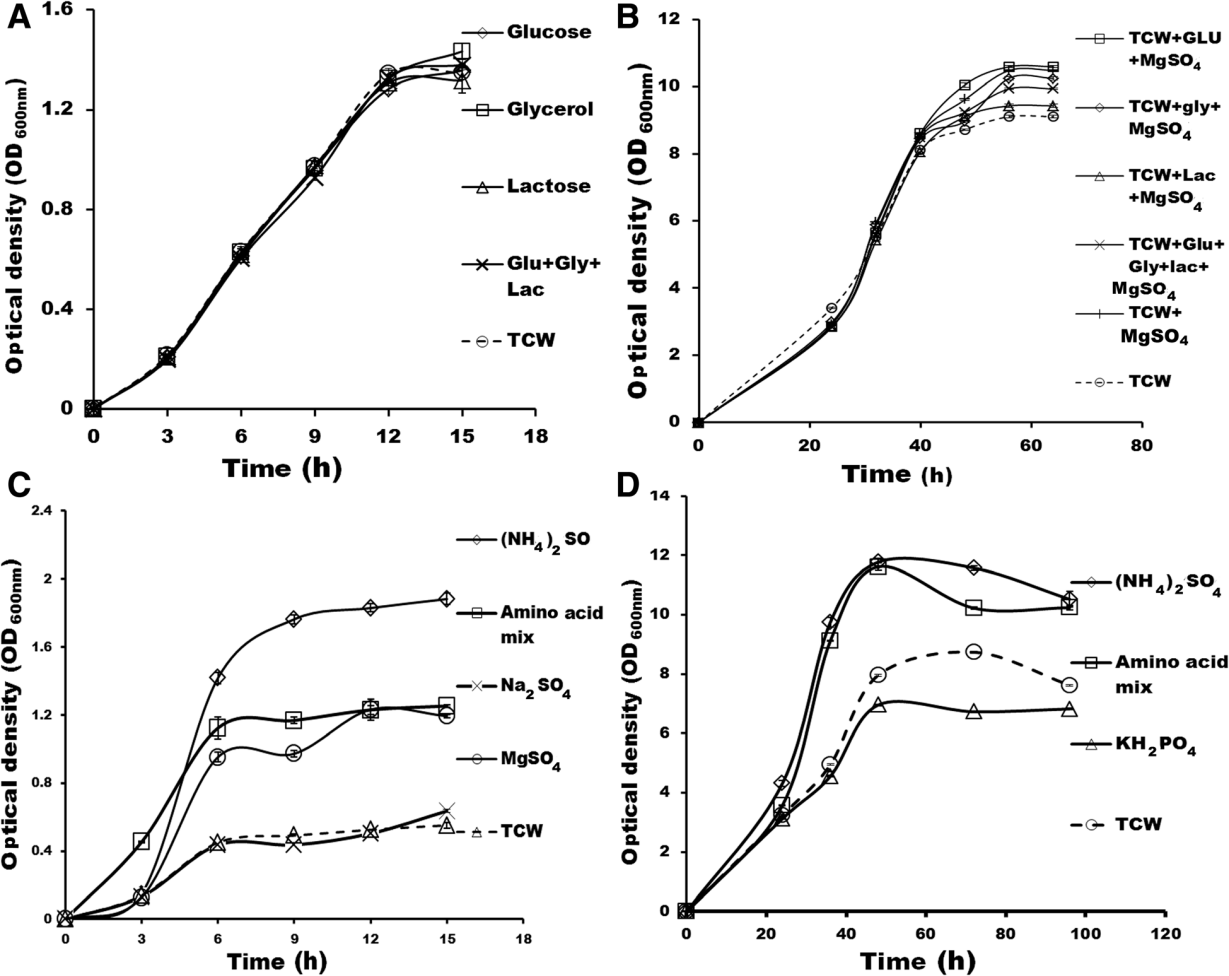
**Growth curve of *E.coli *and *P.pastoris *in TCW-S.** Growth curve of **(A)***E.coli* and **(B)***P.pastoris* in TCW supplemented (TCW-S) with carbon sources (glucose, glycerol and lactose), **(C)** growth curve of *E.coli* and **(D)***P.pastoris* in TCW-S with nitrogen and other components (25 mM NH_4_SO_4_, 0.36% amino acid cocktail, Na_2_SO_4_ 50 mM KH_2_PO_4_ and 2 mM MgSO_4_). The experiments were performed in triplicates and SD has been incorporated.

To achieve consistency in growth rate of *E.coli* and *P.pastoris*, TCW was supplemented by adding carbon, nitrogen and other essential nutrients like magnesium, potassium and sulphur (listed in Table 1). The supplements were chosen based on the work done by Neerathilingam and Markley [21] and Studier [22]. All the compounds were supplemented either individually or in combination. The results were correlated to find the best supplement for TCW. In this regard, the addition of carbon sources such as 0.2% glucose, 0.8% glycerol and 0.5% lactose individually or in combination, did not show any significant improvement in the biomass of *E.coli* as compared to TCW without supplementation (Figure 2A). In case of *P.pastoris*, only slight improvement (1.1 to 1.17 fold) was observed after 60 hours (Figure 2B). This indicates that the limiting factor in TCW is not carbon source but other components.

Furthermore, we supplemented ammonium sulphate, amino acid cocktail, sodium sulphate and magnesium sulphate to TCW. Biomass of *E.coli* was improved by 4 fold in 25 mM (NH_4_)_2_SO_4_ supplemented TCW (1.9 at OD_600nm_), 3 fold in 0.36% amino acid supplemented TCW (1.2 at OD_600nm_) and 2.7 fold in 2 mM MgSO_4_ supplemented TCW (1.1 at OD_600nm_) as compared to TCW without supplementations (0.4 at OD_600nm_) whereas, no considerable improvement was observed for 5mM Na_2_SO_4_ supplementation (Figure 2C). In case of *P.pastoris,* (NH_4_)_2_SO_4_, amino acid cocktail and KH_2_PO_4_ were supplemented to TCW, where biomass improved 1.5 fold in (NH_4_)_2_SO_4_ supplemented TCW (11.6 at OD_600nm_) and 1.4 fold in amino acid cocktail (10.2 at OD_600nm_), as compared to TCW without supplementation (8.7 at OD_600nm_) while, a decreased biomass was observed with 50 mM KH_2_PO_4_ (6.7 at OD_600nm_) (Figure 2D). These results suggest that ammonium sulphate, amino acid and magnesium sulphate supplemented to TCW improves the biomass of both *E.coli* and *P.pastoris* considerably.

**Figure 3:**
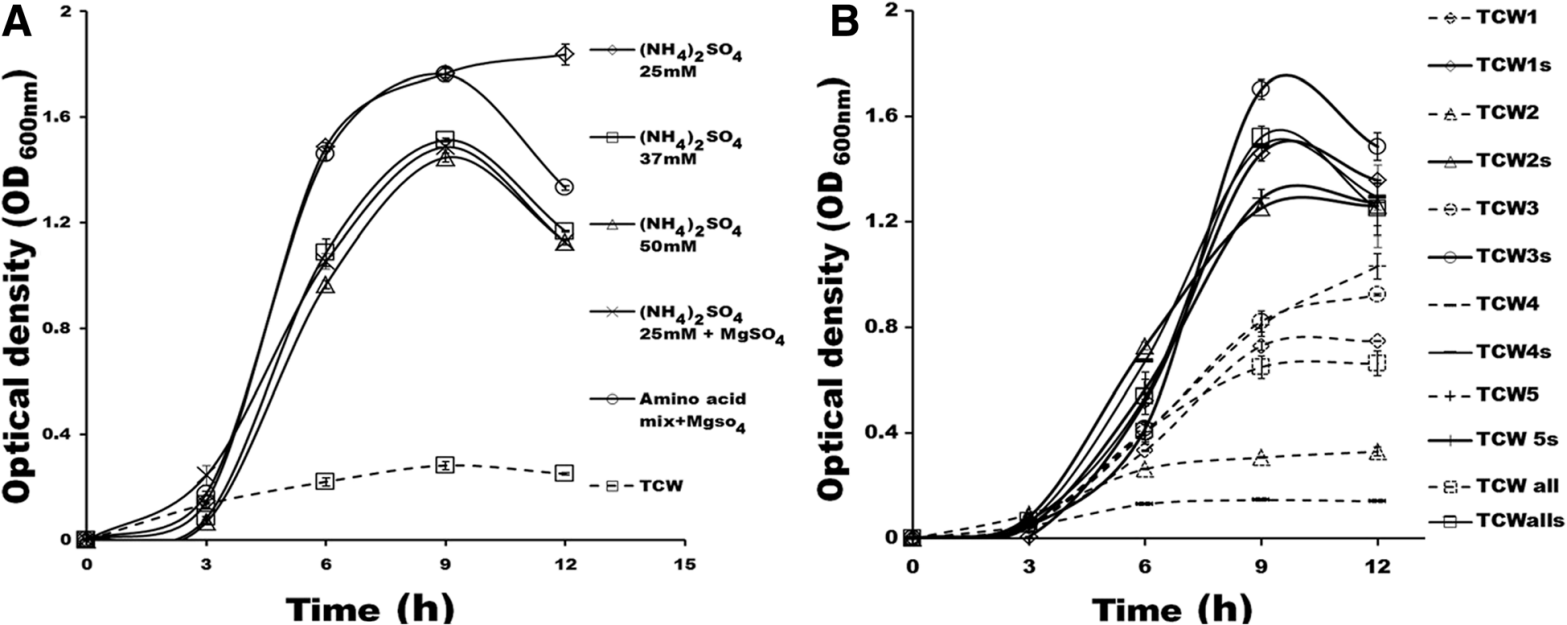
**TCW-S optimization with nitrogen compounds. (A)** Growth curve of *E.coli* in TCW-S supplemented with (NH_4_)_2_SO_4_ at different concentration (25, 37 & 50 mM) and 2 mM MgSO_4_ in combination with 25 mM (NH_4_)_2_SO_4_ and 0.36% amino acid mix. **(B)** (NH_4_)_2_SO_4_ supplementation in five different TCW samples (TCW-S) and comparison with its corresponding TCW. The experiments were performed in triplicates and SD has been incorporated.

In addition, the cumulative effect of supplementation of (NH_4_)_2_SO_4_ and amino acid with MgSO_4_ was noted separately. The biomass of *E.coli* was decreased in (NH_4_)_2_SO_4_+ MgSO_4_ whereas, amino acids + MgSO_4_ supplementation was equivalent to that of the 25 mM (NH_4_)_2_SO_4_. Further to check, increase in concentration of ammonium sulphate improves the biomass of *E.coli,* TCW was supplemented with 25 mM, 37 mM and 50 mM of (NH_4_)_2_SO_4_ and compared with TCW without supplementation. A six fold increase was observed with 25mM (NH_4_)_2_SO_4_ supplementation while only 4.5 fold increase was noted in 37 mM as well as in 50 mM (NH_4_)_2_SO_4_ supplemented TCW (Figure 3A). Above results suggest that the supplementation of 25 mM (NH_4_)_2_SO_4_ is sufficient to achieve the improved and consistent growth.

To validate the ammonium sulphate supplementation for normalizing batch to batch variation of TCW, five samples from different locations were tested. Where, 2–10 fold increase in biomass of *E.coli* in 25 mM (NH_4_)_2_SO_4_ supplemented TCW was observed as compared to TCW without supplementation (Figure 3B). Also, these five samples were pooled together and OD_600nm_ 0.6 was noted while, upon addition of 25 mM (NH_4_)_2_SO_4_ it increased to 1.2. This signifies that the supplementation of TCW with 25 mM (NH_4_)_2_SO_4_ can be used for large scale applications as well, since several coconut fruits would be required to obtain sufficient volume of TCW.

### Carbon and nitrogen estimation of TCW and its correlation with growth

**Figure 4:**
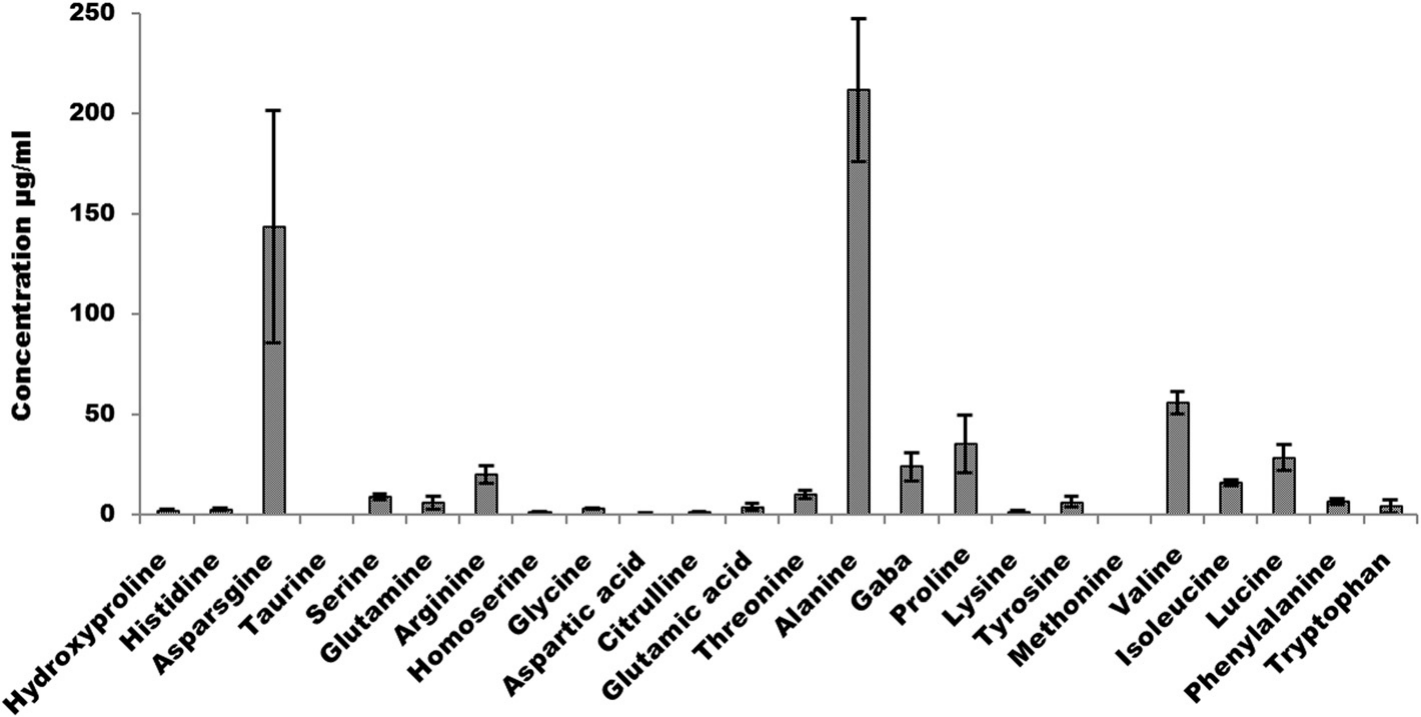
**Mass spectroscopic estimation of TCW.** Estimation of amino acid and metabolites for six different TCW samples was done through LC-MS/SRM method. The median for each compound in these samples was calculated. Variation of amino acids concentration among six different samples of TCW has been indicated as error bar.

To understand the difference in growth pattern and its correlation with nutrient contents of TCW, the chemical profile (carbohydrates, nitrogen and other metabolites) of individual TCW samples used in this study were estimated (Table 2). Phenol sulphuric acid method was adopted to estimate total carbohydrate which ranged from 2.32 to 3.96 g/100 ml in six different TCW samples while, total nitrogen was estimated by Kjeldahl method which fell in the range of 20–60 mg/100 ml. In addition, amino acid and other metabolites were estimated by mass spectroscopy; Asparagine, alanine, valine were present in high concentration (>50 μg/ml) while hydroxyl proline, histidine, taurine, glycine, homoserine, aspartic acid, citrulline, glutamic acid, lysine and methionine were noted in very low concentration (<5 μg/ml) in each sample. Whereas asparagine, alanine, proline, vary vastly among each sample (Standard deviation (SD) >10 μg/ml), while hydroxyl proline, taurine, homoserine, glycine, citrulline, lysine, methionine and aspartic acid showed considerably less variation (SD < 0.5 μg/ml) (Figure 4).

**Table 2:** Chemical profile of TCW used in this study

**Compounds**	**TCW 1 (μg/ml)**	**TCW 2 (μg/ml)**	**TCW 3 (μg/ml)**	**TCW 4 (μg/ml)**	**TCW 5 (μg/ml)**	**TCW 6 (μg/ml)**	SD* (μg/ml)
Hydroxyproline	1.80	2.41	2.52	2.18	2.31	2.39	±0.23
Histidine	2.03	2.57	2.94	1.83	3.61	3.04	±0.60
Asparsgine	176.88	98.07	68.18	97.60	223.48	196.21	±57.92
Taurine	0.07	0.07	0.14	0.09	0.09	0.09	±0.02
Serine	8.27	8.03	8.40	7.01	11.27	10.36	±1.45
Glutamine	6.44	3.32	2.83	5.81	5.11	12.49	±3.17
Arginine	12.49	19.09	17.70	21.49	26.91	22.36	±4.43
Homoserine	1.30	1.39	1.06	1.16	1.31	1.33	±0.11
Glycine	2.90	2.78	2.80	2.39	3.00	3.98	±0.48
Aspartic acid	0.52	0.25	0.50	0.31	0.94	0.71	±0.23
Citrulline	1.02	1.37	1.20	1.10	1.29	1.23	±0.11
Glutamic acid	3.43	1.50	4.93	1.88	6.22	5.00	±1.71
Threonine	10.53	12.25	11.07	5.67	10.63	10.79	±2.08
Alanine	193.56	171.85	252.66	166.57	248.37	237.76	±35.71
Gaba	15.78	14.51	33.24	22.70	30.18	28.18	±7.07
Proline	20.78	56.54	50.26	17.23	31.98	34.73	±14.28
Lysine	0.92	1.27	1.96	1.50	2.14	1.71	±0.41
Tyrosine	6.69	3.48	4.27	4.00	10.32	9.02	±2.61
Methonine	0.03	0.04	0.06	0.16	0.09	0.11	±0.04
Valine	54.69	52.16	48.32	52.79	64.44	62.40	±5.74
Isoleucine	15.48	15.72	14.53	14.89	18.01	17.74	±1.34
Lucine	29.35	24.09	20.21	25.30	38.48	34.71	±6.28
Phenylalanine	7.28	5.03	5.07	4.80	8.66	7.81	±1.53
**Total amino acids metabolites**^**1**^	576.64	498.83	704.50	457.25	759.32	557.02	±107.19
**Total carbohydrate**^**2**^	3.9 g/100 ml	3.82 g/100 ml	2.82 g/100 ml	2.80 g/100 ml	2.32 g/100 ml	2.63 g/100 ml	±0.611 g/100 ml
**Total Nitrogen**^**3**^	33.2 mg/100 ml	17.542 mg/100 ml	33.60 mg/100 ml	15.0 mg/100 ml	57.2 mg/100 ml	28.630 mg/100 ml	±13.78 mg/100 ml

Amino acids and metabolites listed in the table were estimated using mass spectroscopy.

^1^Sum of amino acid and other metabolites concentration estimated by mass spectroscopy.

^2^Total carbohydrate was estimated by phenol sulphuric acid method.

^3^Total nitrogen was estimated by Kjeldahl method.

*SD - standard deviation.

As anticipated from growth patterns of *E.coli* and *P.pastoris* (discussed earlier), the above estimation shows that carbohydrate was present in high concentration with less variation among each samples whereas, total nitrogen showed greater difference (SD > 13 mg/ml). Moreover, in Figure 3B, total nitrogen and amino acid concentration in each six different TCW samples was noted to correlate to its growth pattern. Where the sample with best OD_600nm_ (TCW-5) corresponded to that of highest nitrogen concentration (see Table 2) among others and likewise. Thereby, supporting the assumption discussed earlier, that nitrogen sources are the limiting factor for inconsistent growth rate observed between different TCW samples.

### TCW as an expression media

Many popular bacterial expression system, contain components of the *lac* operon, which can be induced by IPTG. A growth media used for expression of recombinant proteins should not interfere with IPTG induction of the target protein. For example, presence of lactose or high concentration of glucose in the media interferes with the regulation of *lac* operator, which could result in leaky expression or no expression of the target protein [23,24]. Robert and Barbara, demonstrated that addition of 1% glucose to the medium will prevent leaky expression in *lac* based vector systems [24]. Since, TCW is devoid of lactose and also contains ~1% glucose [3], it can be used as a potential media for the expression of recombinant proteins.

**Figure 5:**
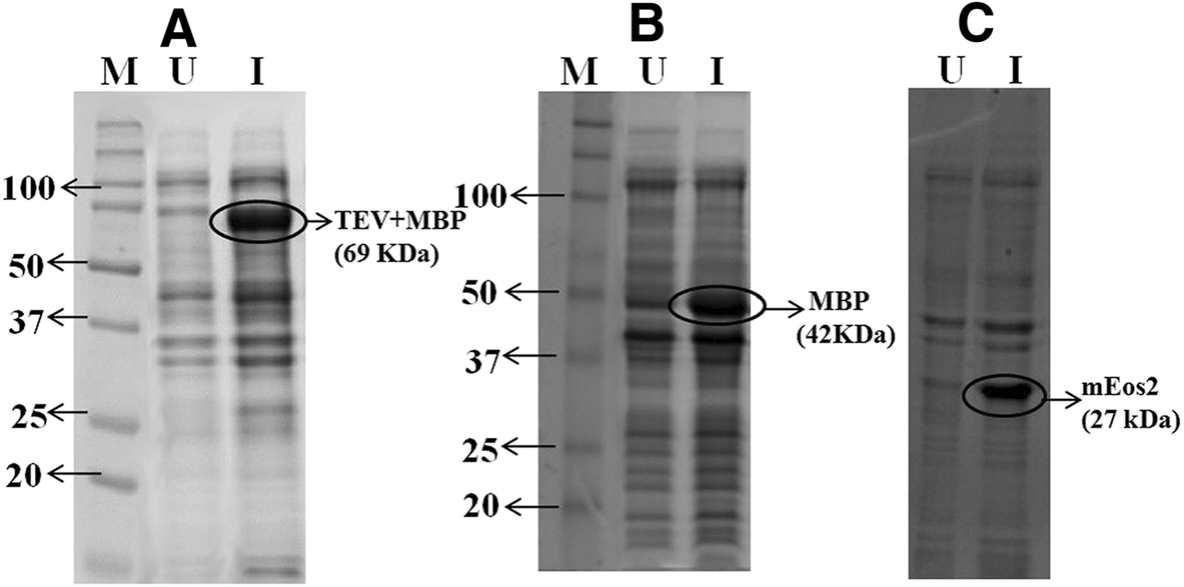
**Recombinant protein expression in *E.coli *grown in TCW media.** Protein expression was analysed in 12% SDS-PAGE. **(A)** MBP-TEV protease expression at 69 KDa harvested after 5 hours **(B)** MBP expressed at 42 KDa harvested after 5 hours **(C)** mEos2 expressed at 27 KDa harvested after overnight induction. All samples normalised based on their OD_600._ Lane M: Marker, Lane U: Uninduced, Lane I: Induced. MBP & MBP-TEV constructs were induced with 0.4 mM IPTG while mEos2 with 0.1 mM IPTG.

**Figure 6:**
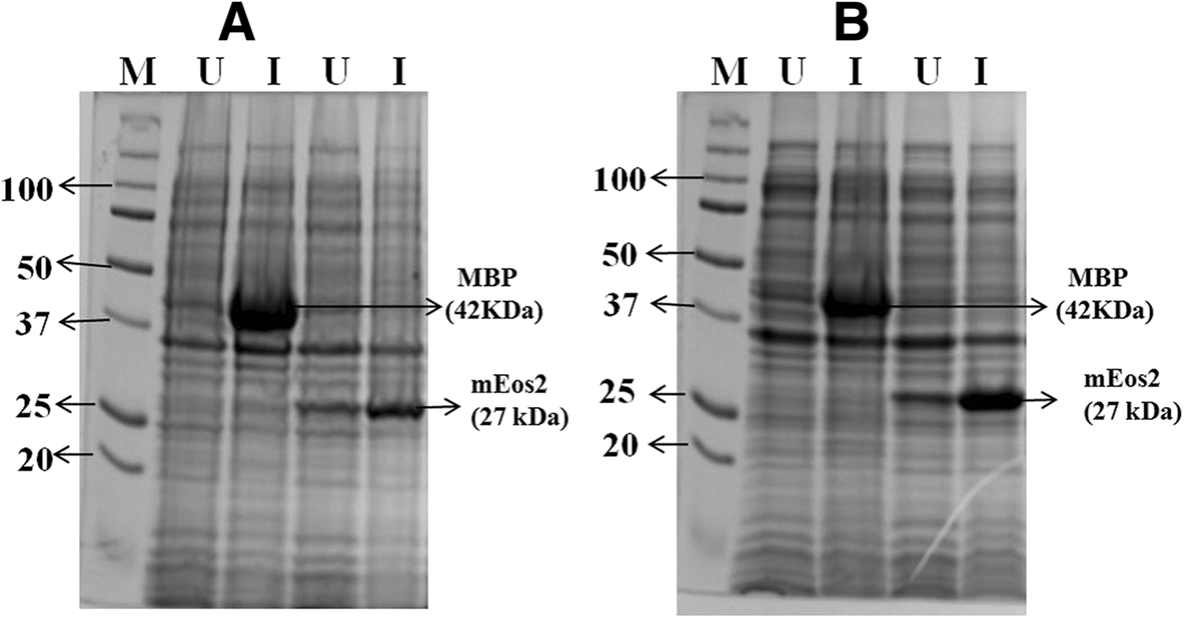
**Recombinant Protein expression in *E.coli *grown in TCW-S.** Protein expression in TCW supplemented with (NH_4_)_2_SO_4_ and its comparison to the expression in LB media. **(A)** MBP (42 KDa) and mEos2 (27 KDa) expression in LB media **(B)** MBP and mEos2 expression in TCW supplemented with (NH_4_)_2_SO_4_. Lane M: Marker, Lane U: Uninduced, Lane I: Induced.

To demonstrate the protein expression in *E.coli* using TCW, three different recombinant proteins were chosen. MBP (42 KDa) is a complex regulatory and transport system used in fusion to the protein of interest to increase the solubility of recombinant proteins [25]. MBP is thus, an easily expressible target in *E.coli*. A longer (69 KDa) construct, which was made from the fusion of MBP and TEV protease (a highly site-specific cysteine protease commonly used for removing affinity tags from purified proteins) was taken as a second construct. The third protein of interest mEoS2 (27 KDa) is a fluorescent protein, which gives a visually detectable green colour ensuring the proteins produced were in the native fold and functional. The constructs were induced using appropriate concentration of IPTG (see Methods), where MBP and MBP- TEV protease was controlled by *tac* promoter (P*tac*) and mEoS2 was controlled by T7 promoter. Successful expression of the above proteins was confirmed by 12% SDS-PAGE (Figure 5). As discussed earlier, biomass of *E.coli* is not consistent in different TCW, which is proportionately related to protein expression. Hence, protein expression was carried out using TCW-S (supplemented with 25 mM (NH_4_)_2_SO_4_). As compared to LB, expression level of mEoS2 was high in TCW-S with less leaky expression. While for MBP, no leaky expression was observed and the expression level was almost equivalent to LB (Figure 6).

Recombinant protein expression in *P.pastoris* is regulated by AOX1 promoter (most widely used), which is induced by methanol; hence the expression media should be devoid of all other carbon sources [26]. TCW contains sugars like glucose, fructose, sucrose etc., which would handicap protein expression. Generally, YPD media is used to generate biomass and is transferred into buffered minimal methanol medium (Invitrogen, USA) for inducing the AOX1 promoter to express the protein of interest. Alternatively, TCW can be used to generate biomass in place of YPD media. If proper optimization for complete utilization of the above mentioned carbon sources is achieved then TCW would be a potential media for protein expression in *P.pastoris* as well.

## Conclusion

Successful expression of MBP, MBP-TEV and mEos2 was observed in TCW and TCW-S, which was comparable that of expression in LB. However, difference in growth rate of *E.coli* and *P.pastoris* was observed, as there was inconsistency of nitrogen source in TCW, which was normalized by the supplementation of TCW with 25 mM (NH_4_)_2_SO_4_. Therefore, the use of TCW alone is not advisable. In order to obtain consistency of growth, 25 mM (NH_4_)_2_SO_4_ supplementation is recommended. In future, other nitrogen sources can be supplemented to obtain higher saturation density in *E.coli* as well as other microbes. Thus, we conclude that TCW can be employed as a natural, inexpensive and efficient growth media for expression of recombinant proteins.

## Competing interests

The authors are co-inventors on a patent application related to the work described in this study, which has been filed through Center for Cellular and Molecular platforms (C-CAMP), Bangalore. The study was financially supported by C-CAMP.

## Authors’ contribution

MN conceptualised and designed the experiments and the study was coordinated by MN, NS and SKV. NS and SKV performed the experiments and were involved in data acquisition. NS, SKV and MN were involved in drafting of manuscript, analysis and interpretation of data. All the authors read and approved the final manuscript.
